# Frozen inactivated autograft replantation for bone and soft tissue sarcomas

**DOI:** 10.3389/fonc.2024.1278237

**Published:** 2024-02-23

**Authors:** Zhichao Tian, Shuping Dong, Yang Yang, Guoxin Qu, Guancong Liu, Xu Liu, Yue Ma, Xin Wang, Weitao Yao

**Affiliations:** ^1^ Department of Bone and Soft Tissue, The Affiliated Cancer Hospital of Zhengzhou University and Henan Cancer Hospital, Zhengzhou, Henan, China; ^2^ Modern Educational Technology Center, Henan University of Economics and Law, Zhengzhou, Henan, China; ^3^ Department of Surgical Department, The Affiliated Cancer Hospital of Zhengzhou University and Henan Cancer Hospital, Zhengzhou, Henan, China

**Keywords:** frozen inactivated, liquid nitrogen, autograft, replantation, osteosarcoma, sarcoma

## Abstract

**Background:**

The frozen inactivation of autologous tumor bones using liquid nitrogen is an important surgical method for limb salvage in patients with sarcoma. At present, there are few research reports related to frozen inactivated autograft replantation.

**Methods:**

In this study, we retrospectively collected the clinical data of patients with bone and soft tissue sarcoma treated with liquid nitrogen-frozen inactivated tumor bone replantation, and analyzed the safety and efficacy of this surgical method. The healing status of the frozen inactivated autografts was evaluated using the International Society of Limb Salvage (ISOLS) scoring system. Functional status of patients was assessed using the Musculoskeletal Tumor Society (MSTS) scale.

**Results:**

This study included 43 patients. The average length of the bone defect after tumor resection is 16.9 cm (range 6.3–35.3 cm). Patients with autograft not including the knee joint surface had significantly better healing outcomes (ISOLS scores) (80.6% ± 15% *vs* 28.2% ± 4.9%, P<0.001) and limb function (MSTS score) (87% ± 11.6% *vs* 27.2% ± 4.4%, P<0.001) than patients with autografts including the knee joint surface. The healing time of the end of inactivated autografts near the metaphyseal was significantly shorter than that of the end far away from the metaphyseal (9.8 ± 6.3 months *vs* 14.9 ± 6.3 months, P=0.0149). One patient had local recurrence, one had an autograft infection, five (all of whom had an autograft including the knee joint surface) had joint deformities, and seven had bone non-union.

**Conclusion:**

Frozen inactivated autologous tumor bone replantation is safe and results in good bone healing. But this method is not suitable for patients with autograft involving the knee joint surface.

## 1 Introduction

Although the incidence of osteogenic sarcoma or malignant tumors of the limbs that invade the bone is low, there are still tens of thousands of newly diagnosed cases worldwide every year (1). Historically, the treatment of bone-derived malignant tumors in the limbs has relied on simple amputation surgery. With the development of surgical techniques and the assistance of neo/adjuvant chemotherapy, the limb salvage rate of bone-derived malignant tumors can reach > 80% (2). Limb salvage surgery for bone-derived malignant tumors usually requires substitutes to repair bone voids following tumor resection and restore limb function. The currently available substitutes include artificial prostheses, autologous bone grafts, allogeneic bone grafts, and inactivated replantation of tumor bone (3, 4).

Inactivation and replantation of tumor bone refer to the removal of an entire block of bone eroded by a tumor (usually malignant or invasive), using various physical or chemical methods to inactivate it *in vitro* and then implanting it *in situ* (4). Compared to other limb salvage surgery methods, tumor bone inactivation and replantation have unique application values, including good matching of *in situ* replantation, no rejection reaction, and low cost (5). Therefore, this surgical method will be indispensable in the foreseeable future.

Tumor bone inactivation methods include pasteurization, microwave, ethanol, *in vitro* irradiation, and freezing (6–8). The use of liquid nitrogen to freeze and inactivate tumor bone has a clinical application history of over 20 years (9). This method involves immersing tumor bones in liquid nitrogen and using ultra-low temperatures of -196°C to destroy tumor cells. Freezing causes tumor cells to lose their activity by inducing ice crystal formation and cell dehydration. Only one cycle of -196°C for 20 minutes is sufficient to kill all tumor cells (10, 11). At present, there are not many research reports related to frozen inactivated autograft replantation. At a center for the diagnosis and treatment of bone and soft tissue sarcoma in a province with a population of nearly 100 million, we used frozen inactivated autograft replantation to treat bone-derived malignant tumors in our clinical work. In this study, we retrospectively collected the clinical data of these patients and reported the treatment efficacy and complications of this surgical method to provide a reference for relevant research and clinical treatment.

## 2 Methods

### 2.1 Patients and eligibility criteria

We retrospectively collected the clinical data of patients who met the inclusion criteria and received treatment at the investigator’s hospital between January 2016 and May 2022. The inclusion criteria were as follows: 1) pathological confirmation of bone and soft tissue sarcoma, 2) received frozen inactivated tumor bone replantation and reconstruction surgery, and 3) complete follow-up data.

This study was reviewed and approved by the hospital’s Medical Ethics Committee, and all treatments and research contents followed the principles of medical ethics.

### 2.2 Treatment protocol

All pathological diagnoses were confirmed by biopsy. Except for the patients with chondrosarcoma, all patients received preoperative and postoperative chemotherapy.

The resection length was determined based on preoperative magnetic resonance imaging. The tumor and surrounding normal tissue were removed as a whole, with a minimum edge of 2 cm. The epiphyseal plate was sacrificed if necessary. Any attached soft tissues or gross tumors were removed from the excised bone, and the bone canals were scraped. The tumor bone was frozen and inactivated using liquid nitrogen. In simple terms, after the extracted tumor bone was rinsed with normal saline, the bone was soaked in liquid nitrogen for 30–40 minutes and then thawed at room temperature (24–26°C) for 15–30 minutes. Frozen inactivated autogenous bone was implanted *in situ* into the bone defect and fixed with a locking plate. In some cases, intercalary grafts of autogenous fibula without a blood supply were used to enhance the strength of the inactivated bone.

Patients were encouraged to immediately begin a moderate range of exercises (whichever is painless) postoperatively. Partial weight-bearing was allowed one month after surgery. Only when a strong bone bonding is achieved is full weight-bearing allowed. Radiological evidence of bone connection at the osteotomy site includes blurred osteotomy lines or sufficiently bridging callus at the host-graft junction.

### 2.3 Collection and evaluation of clinical data

The baseline characteristics of all patients enrolled in this study were reviewed. Bone healing was evaluated using radiography every 3 months until the autograft healed. Follow-up was performed every 3 months in the first year and then every 6 months to check for recurrence and metastasis. We collected data on bone healing, limb function, and complications in these patients after undergoing frozen inactivated autograft replantation, as well as the relationship between their clinical characteristics, bone healing, and limb function.

In this study, the healing status of frozen inactivated autografts was evaluated using the International Society of Limb Salvage (ISOLS) scoring system, which is based on the scores for different parameters (fusion, resorption, fracture, graft shortening, fixation, subluxation, joint narrowing, and subchondral Bone) (12). A score of 100% indicated complete healing of the autograft, whereas a score of 0% indicated complete failure of autograft healing. We defined bone non-union as a condition in which one or both ends of the autograft had not yet fused 12 months after surgery until the fracture or follow-up deadline. Functional status was assessed using the Musculoskeletal Tumor Society (MSTS) scale, which is based on six parameters (pain, functional activity, emotional acceptance, use of external support, walking ability, and gait) (13). A score of 100% indicated a complete recovery of limb function, whereas a score of 0% indicated a complete loss of limb function.

### 2.4 Statistical analysis

Statistical analyses were performed using the SPSS software (version 21.0; IBM, Armonk, NY, USA). Quantitative variables were presented as numerical values (percentages), medians (ranges), or medians (interquartile range). A t-test was used to compare the differences in quantitative variables between the groups. All statistical analyses were two-sided, and a P value <0.05 was considered statistically significant. The follow-up data for this study were obtained as of May 31, 2023.

## 3 Results

The study included 43 patients who met the inclusion criteria (**Tables 1**, **2**). Among them, there were 27 males and 16 females, with an average age of 22.6 (range 9–65) years. There were 30 patients with osteosarcoma, seven with Ewing’s sarcoma, three with chondrosarcoma, one with malignant peripheral nerve sheath tumor, one with round cell sarcoma, and one with myxofibrosarcoma. Most patients had lesions in the femur, followed by the tibia, humerus, radius, and ilium.

**Table 1:** Clinical characteristics of patients who underwent frozen autograft reconstruction.

Patient No.	Gender	Age (year)	Histology	Primary site	Length of autograft (cm)	Contained articular surfaces	Survival	ISOLS score	Bone healing time (months)	Complications	MSTS score
1	Male	20	Round cell sarcoma	Tibia	20.7	No	Yes	92%	F site: 12		100%
2	Female	13	Osteosarcoma	Tibia	12.5	No	No	33%	Not healed	Infection	47%
3	Male	28	Ewing sarcoma	Tibia	14.5	No	No	71%	Not healed		73%
4	Female	17	Ewing sarcoma	Humerus	6.6	No	Yes	79%	C site: 13 F site: Not healed	Bone non-union	67%
5	Female	40	Osteosarcoma	Tibia	13	No	Yes	96%	C site: 12 F site: 21		100%
6	Male	9	Osteosarcoma	Femur	20	Yes	No	25%	F site: 8	Joint deformity	27%
7	Female	9	Ewing sarcoma	Femur	13.5	No	Yes	88%	F site: 12		83%
8	Female	27	Osteosarcoma	Humerus	16	Yes	No	52%	Not healed	Recurrence	77%
9	Female	13	Osteosarcoma	Tibia	24.2	Yes	Yes	33%	F site: 14	Joint deformity	33%
10	Male	21	Osteosarcoma	Humerus	15	Yes	No	83%	Not healed		63%
11	Male	17	Osteosarcoma	Tibia	19.2	No	Yes	79%	C site: 8 F site: 27	Bone non-union	70%
12	Male	15	Osteosarcoma	Tibia	13.1	No	Yes	96%	F site: 8		97%
13	Male	48	Malignant peripheral nerve sheath tumor	Tibia	15	No	Yes	92%	F site: 10		93%
14	Male	18	Osteosarcoma	Radius	13.8	No	Yes	96%	C site: 15 F site: 15		97%
15	Male	17	Osteosarcoma	Tibia	26.4	No	Yes	88%	F site:12		90%
16	Female	15	Ewing sarcoma	Femur	18	No	Yes	63%	C site: 6 F site: not healed	Bone non-union	93%
17	Female	13	Osteosarcoma	Femur	20.5	Yes	No	33%	F site: 9	Joint deformity	23%
18	Female	14	Osteosarcoma	Femur	22.5	No	Yes	83%	F site: 15		93%
19	Male	22	Osteosarcoma	Tibia	13.5	Yes	Yes	28%	C site: 7 F site: 27	Joint deformity	30%
20	Male	11	Osteosarcoma	Tibia	19	No	No	71%	Not healed		87%
21	Male	15	Osteosarcoma	Femur	12.5	No	No	75%	Not healed		83%
22	Male	65	Chondrosarcoma	Ilium	9	No	Yes	92%	C site: 9 F site: 9		90%
23	Female	63	Myxofibrosarcoma	Tibia	8	No	No	79%	Not healed		90%
24	Female	65	Chondrosarcoma	Femur	25.9	No	Yes	96%	C site: 9 F site: 18		93%
25	Male	16	Ewing sarcoma	Femur	18.4	No	Yes	71%	C site: 10 F site: Not healed	Bone non-union	87%
26	Male	12	Osteosarcoma	Femur	16	No	No	92%	C site: 7 F site: 12		97%
27	Male	18	Osteosarcoma	Humerus	19	Yes	No	67%	Not healed		70%
28	Male	17	Ewing sarcoma	Femur	17	No	No	88%	C site: 8 F site: 21		93%
29	Female	10	Osteosarcoma	Tibia	18.2	No	Yes	96%	C site: 7 F site: 13		100%
30	Female	11	Osteosarcoma	Femur	11.8	No	Yes	75%	C site: 8 F site: not healed		87%
31	Male	14	Osteosarcoma	Femur	18.6	No	Yes	92%	C site: 9 F site: 16		97%
32	Male	17	Osteosarcoma	Femur	19	No	Yes	63%	C site: 6 F site: 17		90%
33	Male	32	Osteosarcoma	Humerus	22.4	Yes	Yes	58%	Not healed	Bone non-union	80%
34	Male	21	Osteosarcoma	Femur	20.3	No	Yes	67%	C site: 34 F site: not healed		83%
35	Male	12	Osteosarcoma	Femur	10	No	No	96%	C site: 8 F site: 8		90%
36	Male	27	Osteosarcoma	Femur	17.5	No	Yes	75%	Not healed		90%
37	Female	53	Chondrosarcoma	Femur	20.1	No	Yes	79%	C site: 9 F site: not healed	Bone non-union	87%
38	Male	10	Osteosarcoma	Femur	23.5	Yes	No	22%	Not healed	Bone non-union, Joint deformity	23%
39	Male	48	Osteosarcoma	Tibia	19.5	Yes	No	63%	Not healed		90%
40	Female	14	Ewing sarcoma	Ilium	8	No	Yes	96%	C site: 11 F site: 11		93%
41	Female	12	Osteosarcoma	Femur	13	No	Yes	88%	C site: 7 F site: 30		97%
42	Male	17	Osteosarcoma	Femur	35.3	No	No	92%	C site: 7 F site: 7		90%
43	Male	15	Osteosarcoma	Radius	6.7	No	Yes	100%	C site: 15 F site: 15		100%

ISOLS, International Society of Limb Salvage; MSTS, Musculoskeletal Tumor Society; C site, osteotomy site closer to the epiphysis; F site, osteotomy site far from the epiphysis.

**Table 2:** Pooled clinical characteristics of patients who underwent frozen autograft reconstruction.

Clinical characteristics	Number of patients or value	*P*
Gender		
male	27 (63%)	
female	16 (37%)	
Age (year)	22.6 ± 15.6	
Histology		
osteosarcoma	30 (70%)	
Ewing sarcoma	7 (16%)	
chondrosarcoma	3 (7%)	
others	3 (7%)	
Primary site		
femur	20 (46%)	
tibia	14 (32%)	
humerus	5 (12%)	
radius	2 (5%)	
ilium	2 (5%)	
Length of autograft (cm)	16.9 ± 5.8	
Whether the autograft contained articular surface of the knee joint		
yes	5 (12%)	
no	38 (88%)	
Fixation with plate	43 (100%)	
Average follow-up time (months)	31 (range 10–61)	
Survival		
yes	27 (63%)	
no	16 (37%)	
ISOLS score (%)		<0.001
patients with the autograft containing the knee joint surface	28.2% ± 4.9%	
patients with the autograft not containing the knee joint surface	80.6% ± 15%	
Bone healing time (months)		0.0149
C site	9.8 ± 6.3	
F site	14.9 ± 6.3	
The site of Bone non-union		
C site	0 (0%)	
F site	7 (100%)	
Complications		
bone non-union	7	
joint deformity	5	
infection	1	
recurrence	1	
MSTS score (%)		<0.001
patients with the autograft containing the knee joint surface	27.2% ± 4.4%	
patients with the autograft not containing the knee joint surface	87% ± 11.6%	

Data are presented as percentages (number events/total). ISOLS, International Society of Limb Salvage; C site, osteotomy site closer to the epiphysis; F site, osteotomy site far from the epiphysis; MSTS, Musculoskeletal Tumor Society.

**Figure 1:**
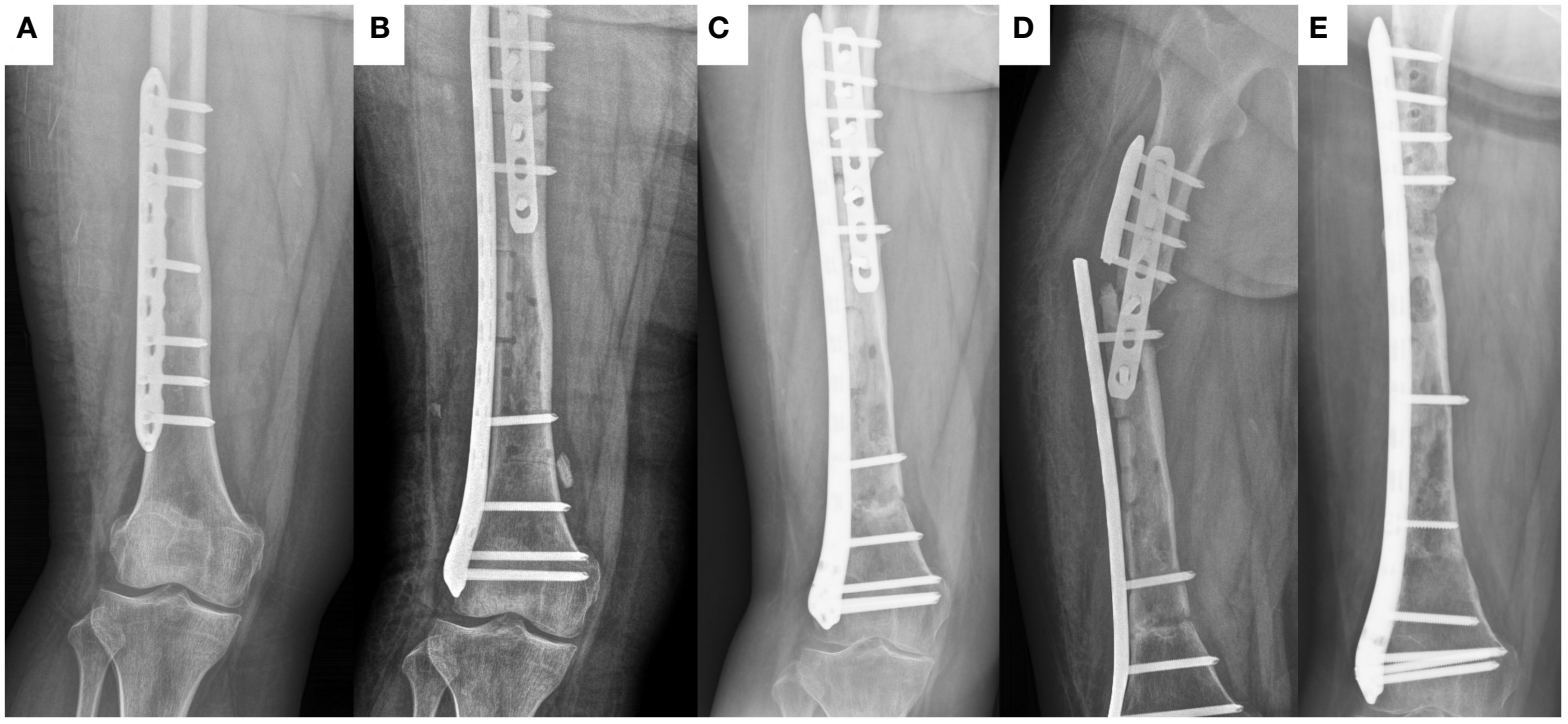
The healing process of frozen inactivated autologous tumor bone in a 15-year old female (case 37) with Ewing sarcoma involving the diaphysis of the left femur. **(A)** preoperative X-ray. **(B)** postoperative X-ray. **(C)** X-ray at 18 months postoperation. **(D)** X-ray at 20 months postoperation (At this point, the plate breaks). **(E)** X-ray at 11 months after last operation.

The average follow-up duration was 31 months (10–61 months). Before the data deadline of this study, 27 (63%) patients were still alive, and 16 (37%) died of sarcoma metastasis. The average length of the bone defect after tumor resection is 16.9 cm (range 6.3–35.3 cm). The inactivated autograft of five patients included the articular surface of the knee joint. All patients underwent steel plate internal fixation. Patients who did not include the knee joint had significantly better healing outcomes (ISOLS scores) (80.6% ± 15% *vs* 28.2% ± 4.9%, P<0.001) and limb function (MSTS score) (87% ± 11.6% *vs* 27.2% ± 4.4%, P<0.001) than those who included the knee joint. The healing time of the end of inactivated autografts near the metaphyseal was significantly shorter than that of the end far away from the metaphyseal (9.8 ± 6.3 months *vs* 14.9 ± 6.3 months, P=0.0149) (**Figure 1**).

**Figure 2:**
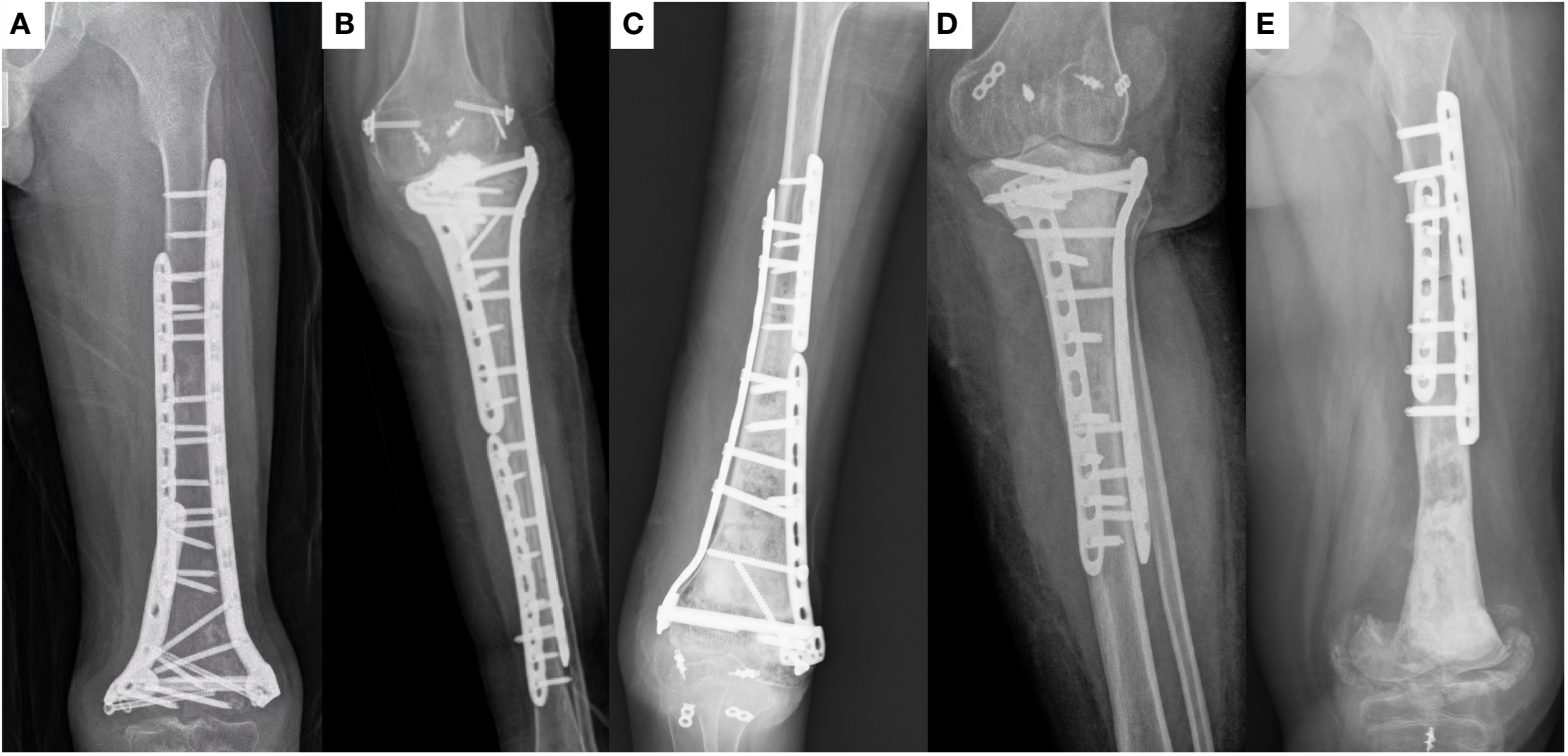
X-ray of patients with joint deformity. All five patients (case 6, 9, 17, 19, and 38, respectively) with inactivated bone containing the knee joint surface experienced this complication.

In terms of complications, one patient had local recurrence, one patient had infection of the autograft, five patients (all of which included the joint surface of the knee) had joint deformities (**Figure 2**), and seven patients had bone non-union (**Figure 1**).

## 4 Discussion

In this study, we retrospectively collected the clinical data of patients with bone and soft tissue sarcomas treated with liquid nitrogen-frozen inactivated tumor bone replantation and analyzed the safety and efficacy of this surgical method. Among the 43 patients included, only one had local recurrence, and one had an autograft infection. Seven patients experienced bone non-unions. Most of the patients achieved bone healing. Bone healing and limb function of patients with autografts not including the knee joint surface were significantly better than those of patients with autografts including the knee joint surface.

The greatest risk of inactivated autologous tumor bone replantation is tumor recurrence due to incomplete inactivation. Historically, the methods used for inactivation include boiling, pasteurization, alcohol, radiation, and liquid nitrogen freezing used in this study (6–8). Among these methods, alcohol inactivation has the highest reported recurrence rate. This is related to the inability of alcohol to penetrate deep into the bone tissue (14). The recurrence rate in this study was relatively low, similar to that in other studies using liquid nitrogen inactivation (9, 15, 16), and similar to the recurrence rates for inactivation by boiling, pasteurization, and radiation (6–8). This demonstrates the safety of liquid nitrogen inactivation. However, there is still no standard for the duration of liquid nitrogen immersion. Our initial surgical plan used soaking for 40 min based on other studies. Subsequent patients were shortened to 30 min. The shortest reported soaking time was 20 min (11, 17). Currently, the shortest safe soaking time remains unknown.

Another major surgical risk associated with inactivation with liquid nitrogen is infection. Some studies have recommended the use of ultraviolet radiation for liquid nitrogen sterilization (16, 18). In this study, we used ordinary liquid nitrogen tank tubes to store liquid nitrogen and specialized containers for high-temperature disinfection to soak the tumor bone in liquid nitrogen. The infection rate in this study was low, suggesting that no additional ultraviolet irradiation was required to sterilize liquid nitrogen.

Difficulty in bone healing is a common problem in inactivated autologous bone replantation (3, 19). Difficulties in bone healing can lead to a series of complications, including non-union, fractures, internal fixation fractures, and long-term bone resorption (16, 19). Many researchers believe that freezing can completely preserve various osteogenic proteins in tumor bone segments. Therefore, similar to radiation inactivation, it is more conducive to bone healing than high-temperature inactivation (7, 16, 20). However, healing of frozen inactivated autografts remains difficult. In this study, we found that the sites with bone non-union were all at the ends of the inactivated autografts far from the metaphyseal region. The ends near the metaphysis healed smoothly (**Figure 1**). This indicates that blood supply around the autograft is very important. Interestingly, inactivated autografts located in the middle of the radius healed smoothly. We speculate that this may be related to moderate pressure on the radius. Two patients (cases 16 and 37) with difficulty in bone healing in the middle of the femur underwent local autologous iliac cancellous bone grafting surgery, and the autograft healed within approximately one year. This indicates that autologous iliac cancellous bone-grafting surgery at the non-union site of inactivated autograft can effectively promote healing. Another noteworthy issue is the effect of age on the healing of inactivated bones. Currently, a small number of studies have focused on this issue, but due to the small sample size, it is not possible to draw definitive conclusions (21, 22). In this study, age did not seem to be a relevant factor for inactivated bone healing. However, due to the limited sample size, we are unable to draw a definitive conclusion here. These findings warrant further in-depth research.

Limb function recovery is an important indicator of the efficacy of inactivated autograft replantation surgery. Inactivated bone has optimal osteogenesis, osteoinduction, osteoconduction, and histocompatibility properties, along with the lower the risk of immunological rejection (3). Prosthetic replacement is an option to provide immediate stability and early weightbearing capability, but the risk of complications, such as loosening, infection, implant breakage and fracture of the adjacent bone may be higher than those for biological reconstructions (9). Therefore, in many cases, inactivated bone replantation can achieve better limb function than prosthesis replacement. This study further confirms this judgment. In this study, the limb function of patients with autograft that did not include the knee joint surface was better than that of patients who have undergone prosthesis replacement surgery (23–25). However, the limb function of patients with autograft that included the knee joint surface was significantly poorer than that of patients with autograft that did not include the knee joint surface. All patients with autograft including the surface of the knee joint experienced severe joint dysfunction (**Figure 2**). Other studies have identified this problem (26, 27). This indicates that inactivated autograft replantation is unsuitable for the treatment of malignant bone tumors involving the knee joint surface. The surgical method of inactivated autograft replantation combined with artificial joints can be used to improve the limb function of these patients (27, 28).

This study had some limitations, including its retrospective nature, small sample size, and lack of a control group. Additionally, this study included a heterogeneous sample with different diagnoses and tumor locations. All these factors make it difficult to analyze the differences in outcomes and complications. More patients and additional studies involving a control group are required to standardize this technique.

In conclusion, Liquid nitrogen-frozen inactivated autologous tumor bone replantation is safe and results in good bone healing. The bone healing and limb function of patients with frozen inactivated autografts that did not include the knee joint surface were significantly better than those of patients with autografts that included the knee joint surface.

## Data availability statement

The original contributions presented in the study are included in the article/supplementary material. Further inquiries can be directed to the corresponding author.

## Ethics statement

The studies involving humans were approved by Medical Ethics Committee of Henan Cancer Hospital. The studies were conducted in accordance with the local legislation and institutional requirements. Written informed consent for participation in this study was provided by the participants’ legal guardians/next of kin. Written informed consent was obtained from the individual(s), and minor(s)’ legal guardian/next of kin, for the publication of any potentially identifiable images or data included in this article.

## Author contributions

ZT: Conceptualization, Data curation, Investigation, Methodology, Validation, Writing – original draft. SD: Data curation, Investigation, Writing – original draft. YY: Data curation, Formal analysis, Methodology, Software, Writing – original draft. GQ: Data curation, Investigation, Writing – original draft. GL: Data curation, Investigation, Writing – original draft. XL: Investigation, Writing – original draft. YM: Investigation, Writing – original draft. XW: Conceptualization, Investigation, Resources, Writing – review & editing. WY: Conceptualization, Resources, Writing – review & editing.
